# Diagnostic accuracy of standard practice wrist MRI for scapholunate ligament injuries: a retrospective arthroscopic correlation study

**DOI:** 10.1007/s00402-026-06541-8

**Published:** 2026-10-20

**Authors:** Sebastian Spodzieja, Julie Boever, Johannes Rau, Thomas Bruckner, Holger Müller, Jan Wulf, Frank Unglaub, Adrian Cavalcanti Kussmaul

**Affiliations:** 1 Department of Hand Surgery, Vulpius Clinic, Bad Rappenau, Germany; 2 Department of Orthopaedics and Trauma Surgery, Musculoskeletal University Center Munich (MUM), Ludwig-Maximilians-Universität München, Munich, Germany; 3 Department of Traumatology, Hand Surgery and Sports Medicine, ViDia Clinics Karlsruhe, Karlsruhe, Germany; 4 Institute of Medical Biometry (IMBI), Heidelberg, Germany; 5 Department of Orthopaedics and Trauma Surgery, Orthopaedic-Trauma Surgery Center, University Medical Centre Mannheim, Medical Faculty Mannheim, Heidelberg University, Mannheim, Germany

**Keywords:** Scapholunate ligament, Wrist arthroscopy, Magnetic resonance imaging, Diagnostic accuracy, Sensitivity, Specificity

## Abstract

**Introduction:**

The scapholunate ligament is the most injured intercarpal ligament. Untreated tears can lead to carpal instability and subsequent radiocarpal and midcarpal osteoarthritis. While wrist arthroscopy remains the gold standard, the diagnostic performance of standard practice preoperative MRI in clinical practice warrants further investigation.

**Methods:**

We retrospectively analysed patients who underwent preoperative wrist MRI followed by wrist arthroscopy between 2013 and 2021. MRI findings regarding scapholunate ligament integrity were compared with arthroscopic findings serving as reference standard. Sensitivity and specificity were calculated for detection of any scapholunate ligament lesion (partial or complete tear).

**Results:**

A total of 889 patients were included. Complete paired data were available for 864 patients with a mean age of 42.4 ± 15.4 years (range 10–83 years). 51.3% were male. MRI classified the ligament as intact in 83.9%, partially ruptured in 9.1%, and completely ruptured in 6.9%. Arthroscopy identified intact ligaments in 78.2%, partial tears in 12.9%, and complete tears in 8.9%. In this cohort, MRI demonstrated high specificity (91.1%, 95% CI 89.0–93.3) but limited sensitivity (42.0%, 95% CI 35.0–49.1) for scapholunate ligament lesions.

**Conclusion:**

In standard clinical practice, preoperative wrist MRI shows high specificity but insufficient sensitivity for detecting scapholunate ligament injuries. In this cohort, MRI detected intact ligaments reliably, but a relevant proportion of partial and complete tears remained unrecognized.

## Introduction

 The scapholunate interosseous ligament (SLIL) is the most commonly injured carpal ligament, with tears representing a leading cause of chronic wrist pain and dynamic or static instability [1–4]. Typical causes are hyperextension injuries of the wrist or repetitive trauma, such as prolonged use of crutches [5]. Diagnostic evaluation begins with a detailed history, clinical examination, and standard radiographs. Clinically, partial and complete ruptures are distinguished and further categorized arthroscopically according to the Geissler classification [6]. Untreated SLIL injuries can progress through a predictable cascade of carpal malalignment, culminating in scapholunate advanced collapse (SLAC) wrist with radiocarpal and midcarpal osteoarthritis [1, 3, 7]. Early and accurate diagnosis is therefore essential to prevent irreversible degenerative changes [1].

Wrist arthroscopy remains the diagnostic gold standard, providing direct visualization and Geissler grading of ligament integrity under dynamic loading conditions [1, 6]. However, its invasiveness has driven widespread adoption of magnetic resonance imaging (MRI) as the primary non-invasive imaging modality for intrinsic wrist ligament assessment [8, 9].

However, reported diagnostic accuracy varies widely, with standard practice clinical MRI often demonstrating limited sensitivity (19–79%) for SLIL tears despite reasonable specificity (69–98%) [5, 9, 10]. Notably, partial tears are consistently underdetected compared to complete ruptures [8]. Factors influencing MRI accuracy include field strength (1.5 T vs. 3 T), dedicated wrist coils, contrast medium administration, and radiological musculoskeletal expertise [11–14]. Large-scale data from standard clinical imaging—performed under variable conditions without standardized research protocols—remain scarce.

The purpose of this retrospective study was to assess the diagnostic accuracy of standard practice preoperative wrist MRI for SLIL lesions in a large clinical cohort using wrist arthroscopy as reference standard, and to determine whether contemporary practice has improved upon previously reported benchmarks of sensitivity and specificity.

## Methods

### Study design and patient selection

We reviewed all consecutive patients who underwent preoperative wrist MRI followed by diagnostic and/or therapeutic wrist arthroscopy at the Vulpius clinic in Bad Rappenau, Germany between January 2013 and December 2021.

Patients who underwent preoperative wrist MRI and had the original MRI report available were included in the study. Exclusion criteria were MRI performed after arthroscopy, MRI examinations performed at 3 T, an interval of more than 1 year between MRI and arthroscopy, prior SLIL surgery, surgery not performed by a board-certified hand surgeon, and incomplete medical records.

### Data collection

**Fig. 1:**
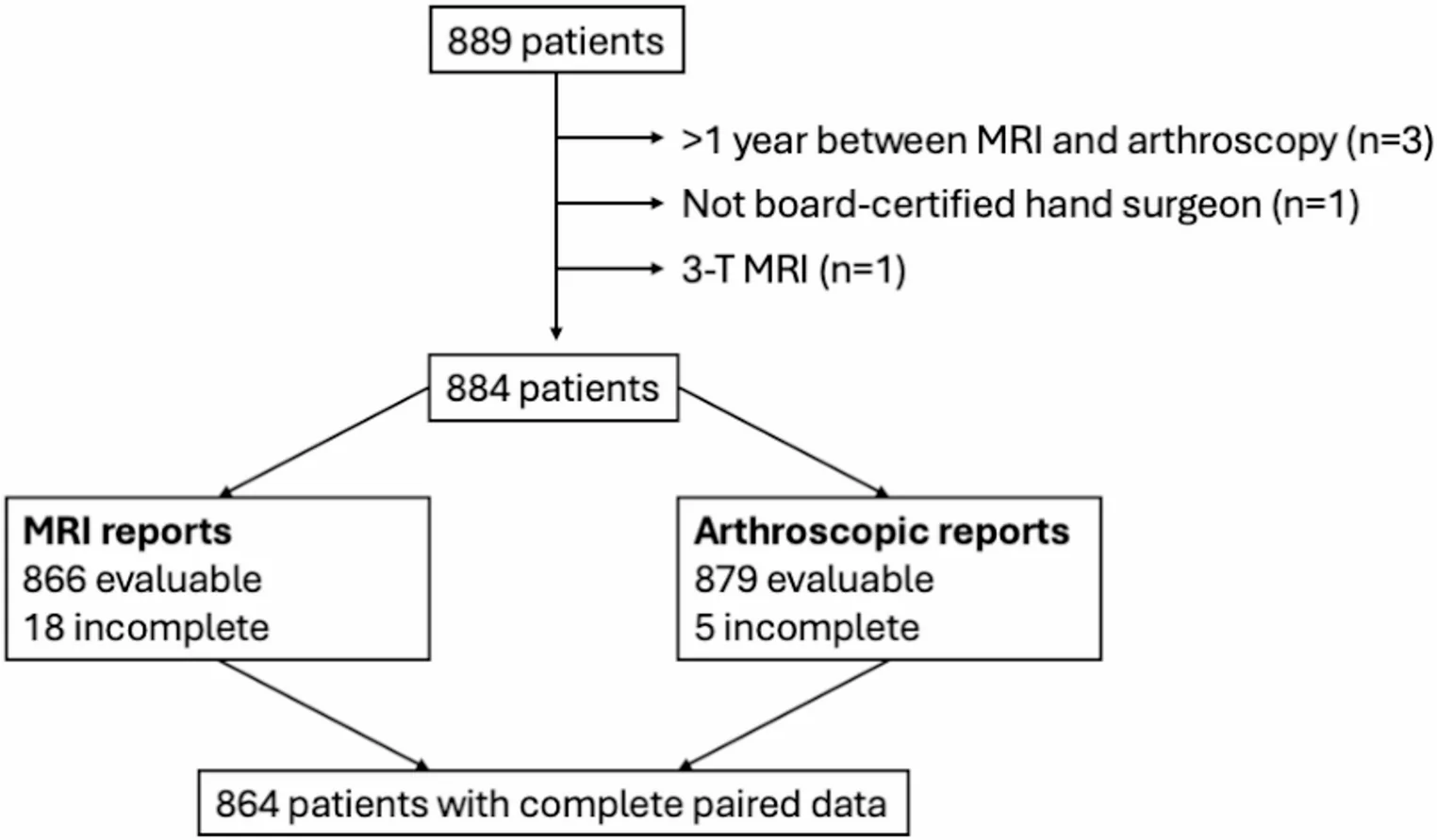
Complete paired data of 864 patients

Patient demographics (age, sex, operated side), MRI technical details (contrast medium use, dedicated wrist coil/field strength documentation), and time interval between MRI and surgery were extracted from medical records. MRI reports were reviewed for explicit mention of SLIL status, categorized as: intact, partial tear (Geissler grades I-III), or complete tear (Geissler grade IV). Arthroscopic findings were recorded according to the Geissler classification by the operating surgeon. No attempts were made to retrieve missing information from the examiners to ensure an unbiased assessment of MRI report quality. All reports without explicit mention of the scapholunate ligament were excluded (see Fig. 1).

### Surgical technique

Wrist arthroscopy was performed under regional anaesthesia using standard 3/4 and 4/5 or 6R portals with traction (mean 3 kg) in dry technique. Both radiocarpal and midcarpal compartments were systematically inspected. SLIL integrity was assessed dynamically using a probe, with grading according to Geissler et al.: Grade I (attenuation/haemorrhage), Grade II (partial tear, probe passes < 1 mm gap), Grade III (incomplete tear, probe passes gap), Grade IV (complete tear, drive-through sign) [6]. Partial tears (Grades I-III) were grouped together for primary analysis. All procedures were performed or supervised by three experienced board-certified hand surgeons.

### Statistical analysis

Continuous variables were reported as mean ± standard deviation (SD). Categorical variables were presented as frequencies and percentages. Sensitivity, specificity, positive predictive value (PPV), and negative predictive value (NPV) were calculated using a 2 × 2 contingency table, with arthroscopy as reference standard. Any SLIL lesion (partial or complete) on MRI versus arthroscopy constituted the primary endpoint. Subgroup analyses examined the impact of contrast or coil use. Kappa values were interpreted as follows: slight agreement K = 0-0.20; fair agreement K = 0.21–0.40; moderate agreement K = 0.41–0.60; substantial agreement K = 0.61–0.80; and almost perfect agreement K = 0.81-1.00. Statistical significance was set at *p* < 0.05. Analyses were performed using SAS version 9.4 WIN (SAS Institute, Cary, NC, USA).

## Results

### Patient characteristics

A total of 889 patients were included in the analysis. Complete paired data were available for 864 patients (Fig. 1) with a mean age of 42.4 ± 15.4 years (range, 10–83 years) and a mean interval between MRI and arthroscopy of 51.5 ± 44.0 days (range, 1–295 days). Males comprised 51.3% (443/864) of the cohort, and the right wrist was operated in 56.9% (492/864) of cases. Most procedures were performed by surgeon A (55.4%, 479/864), followed by surgeon B (33.7%, 291/864) and surgeon C (10.9%, 94/864).

### MRI and arthroscopic findings

In the MRI reports the scapholunate interosseous ligament (SLIL) was classified as intact in 725 cases (83.9%), partially torn in 79 (9.1%), and completely torn in 60 (6.9%) (Table 1). MRI technical details revealed contrast medium use in 21.3% and dedicated wrist coil in 37.7%.

Arthroscopic findings showed intact ligaments in 676 (78.2%), partial tears in 111 (12.9%), and complete tears in 77 (8.9%) (Table 1).

**Table 1:** SLIL findings in MRI and during arthroscopy (*n* = 864)

SLIL status MRI ↓ Arthroscopy →	Intact	Partial tear (Geissler I-III) [6]	Complete tear (Geissler IV) [6]	Total
Intact	616 (71.3%)	76 (8.8%)	33 (3.8%)	725 (83.9%)
Partial tear (Geissler I–III) [6]	43 (5.0%)	27 (3.1%)	9 (1.0%)	79 (9.1%)
Complete tear (Geissler IV) [6]	17 (2.0%)	8 (0.9%)	35 (4.1%)	60 (6.9%)
Total	676 (78.2%)	111 (12.9%)	77 (8.9%)	864 (100.0%)

### Diagnostic performance in paired analysis

The contingency table demonstrated limited agreement between modalities (McNemar’s test: χ²=14.33, df = 3, *P* = 0.0025). Simple kappa was 0.339 (95% CI: 0.269–0.409) and weighted kappa was 0.399 (95% CI: 0.324–0.475), indicating only fair agreement.

When dichotomized (any lesion vs. intact), MRI showed a sensitivity of 42.0% (95% CI: 35.0–49.1), specificity of 91.1% (95% CI: 89.0–93.3), PPV of 56.8% (95% CI: 48.6–65.1), and NPV of 85.0% (95% CI: 82.4–87.6) (McNemar’s test: χ²=14.21, *P* = 0.0002) (Table 2).

**Table 2:** Contingency table MRI vs. arthroscopy

SLIL Status MRI ↓ Arthroscopy →	Intact	Any lesion	Total
Intact	616 (71.3%)	109 (12.6%)	725 (83.9%)
Any lesion	60 (6.9%)	79 (9.1%)	139 (16.1%)
Total	676 (78.2%)	188 (21.8%)	864 (100%)

Any lesion=Geissler I–IV

When lesions were dichotomized excluding partial lesions, MRI demonstrated a sensitivity of 51.5% (95% CI: 39.6–63.4) and a specificity of 97.3% (95% CI: 96.1–98.6). The positive predictive value (PPV) was 67.3% (95% CI: 54.6–80.1), and the negative predictive value (NPV) was 94.9% (95% CI: 93.2–96.6) (Table 3).

**Table 3:** Contingency table MRI vs. arthroscopy (dichotomized, excluding partial lesions)

SLIL status MRI ↓ arthroscopy →	Intact	Complete lesion	Total
Intact	616 (87.9%)	33 (4.7%)	649 (92.6%)
Complete lesion	17 (2.4%)	35 (5.0%)	52 (7.4%)
Total	633 (90.3%)	68 (9.7%)	701 (100%)

### Subgroup analyses

**Fig. 2:**
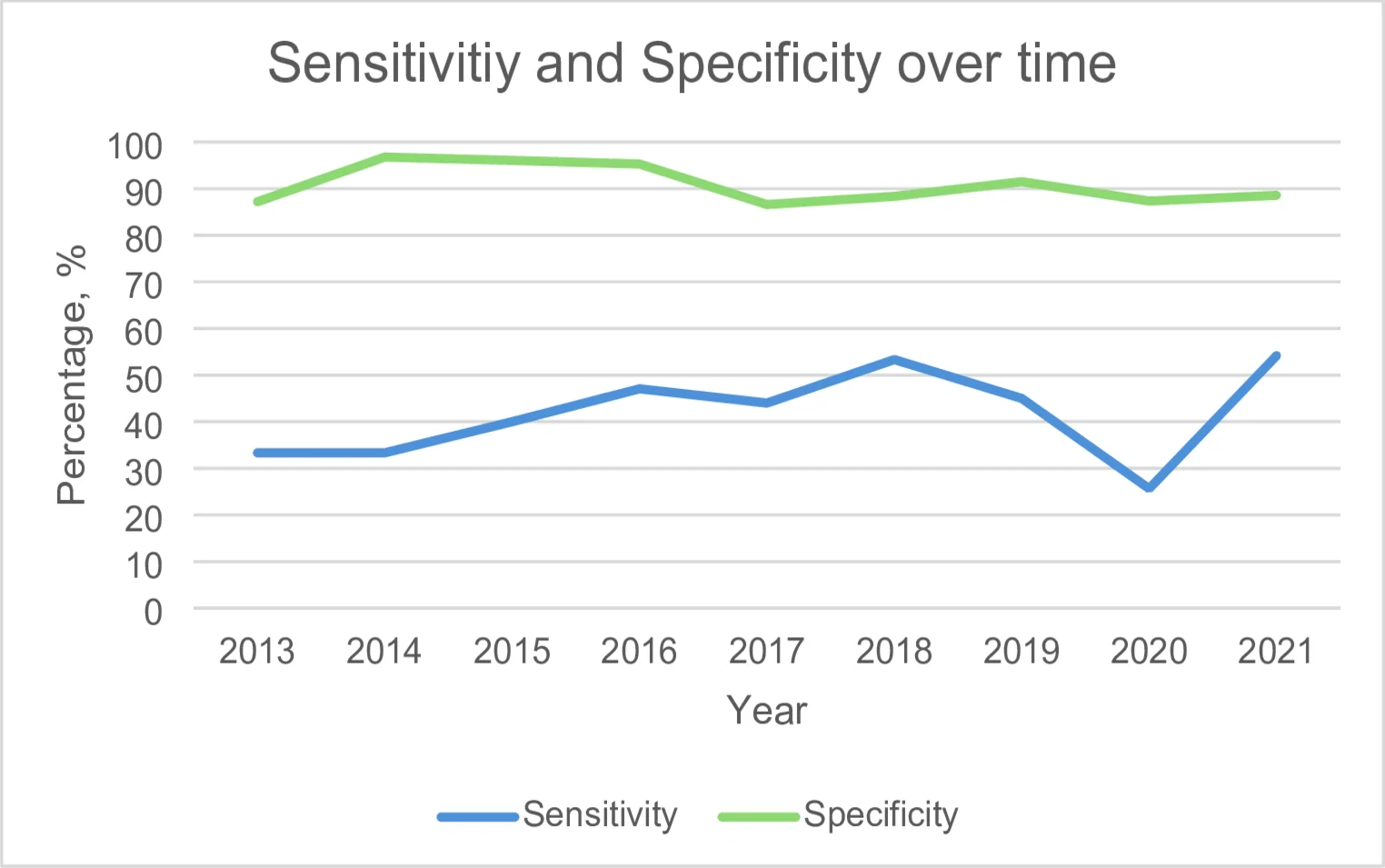
Sensitivity and specificity of MRI from 2013 to 2021

Sensitivity of MRI increased over time from 33.3% (2013) to 54.2% (2021), while specificity remained stable (86.4–96.8%) (Fig. 2).

By MRI technique, sensitivity was higher without documented wrist coil than with coil (48.3% vs. 32.4%) and higher with contrast than without (50.0% vs. 39.4%). The highest sensitivity (66.7%) occurred without wrist coil but with contrast (*n* = 106), though numbers were small (Table 4).

**Table 4:** Sensitivity by MRI characteristics

Subgroup	Sensitivity, % (95% CI)	Specificity, % (95% CI)	*n*
No coil	48.3 (39.1–57.4)	89.4 (86.5–92.3)	538
With coil	32.4 (21.8–43.1)	94.1 (91.1–97.0)	326
No contrast	39.4 (31.4–47.5)	91.8 (89.5–94.1)	680
With contrast	50.0 (35.6–64.5)	88.4 (83.1–93.8)	184
No coil, no contrast	43.3 (33.1–53.6)	91.2 (88.2–94.2)	432
No coil, with contrast	66.7 (47.8–85.5)	81.7 (73.3–90.1)	106
With coil, no contrast	32.7 (19.9–45.4)	92.9 (89.3–96.5)	248
With coil, with contrast	31.8 (12.4–51.3)	98.3 (94.8–100)	78

## Discussion

This study demonstrated that standard practice preoperative wrist MRI has high specificity but only limited sensitivity for detecting scapholunate interosseous ligament lesions when compared with arthroscopy. The discrepancy between standard practice MRI reports and arthroscopic findings suggests that false-negative MRI interpretations are not seldom in SLIL injury, especially in partial lesions, which may partly explain the limited sensitivity observed in standard clinical practice [8]. In this large cohort, MRI correctly identified many intact ligaments, but a substantial proportion of partial and complete tears were missed. Notably, specificity and negative predictive value further increased when partial lesions were excluded, whereas sensitivity remained only moderate, underscoring the limited ability of MRI to reliably rule out clinically relevant ligament injuries. Similar diagnostic limitations of standard practice MRI have been reported in contemporary studies, including a 2020 analysis showing an overall MRI sensitivity of 38.5% and specificity of 91.0% for SLIL injuries [9]. These findings support the role of MRI as a useful initial non-invasive test, but not as a standalone modality for excluding clinically relevant SLIL pathology [15]. Notably, compared with the standard clinical setting reported by Mayer et al. in 2013, in which MRI achieved a sensitivity of 32% and specificity of 98% for SLIL tears, our cohort indicates only a modest gain in sensitivity, while specificity remains similarly high; thus, standard practice MRI still cannot reliably exclude clinically relevant SLIL injury [5].

**Fig. 3:**
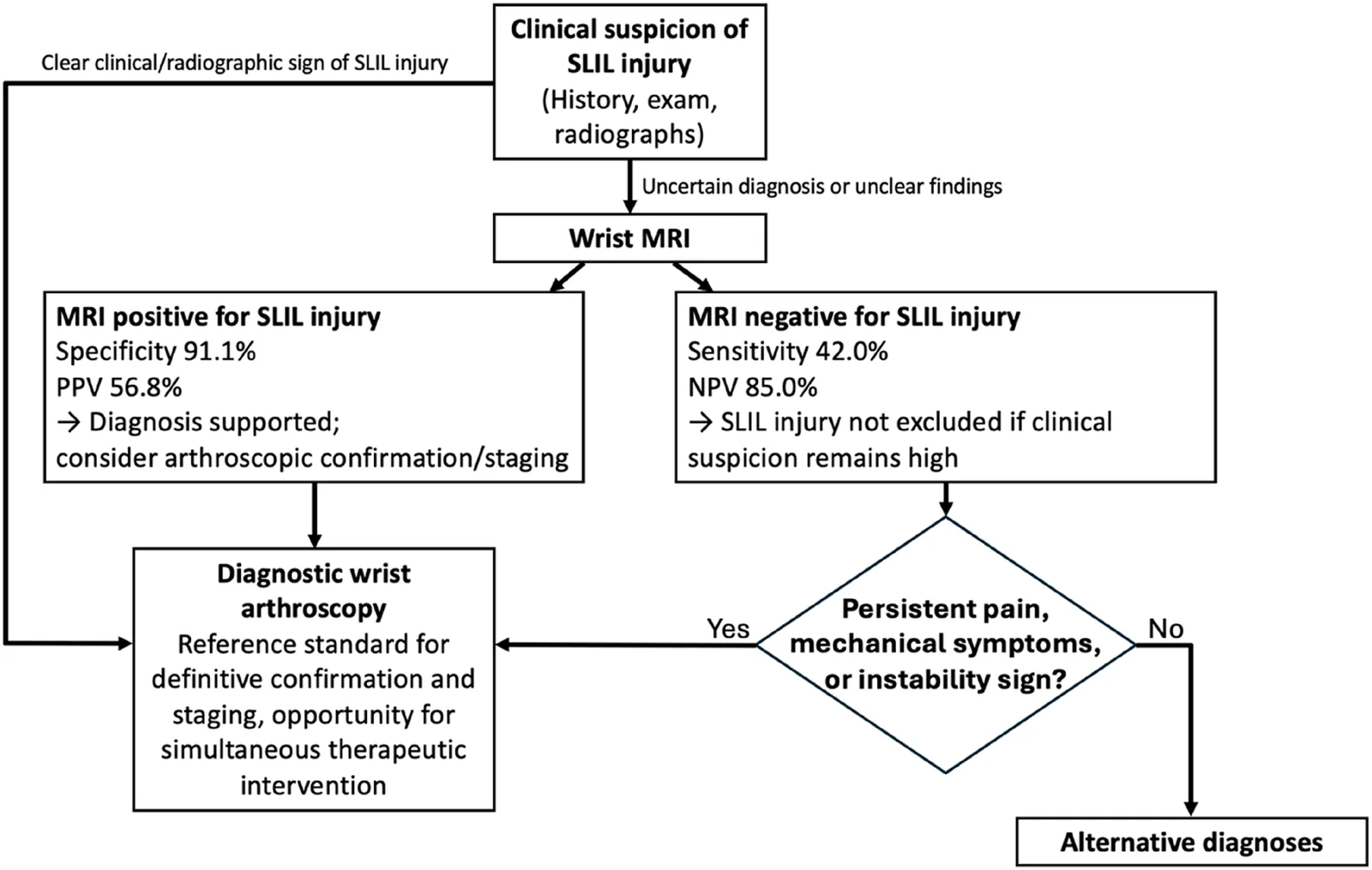
Suggested management pathway for suspected SLIL injury. Diagnostic accuracy measures (sensitivity, specificity, PPV, and NPV) are derived from our cohort. Although a positive MRI supports the diagnosis, wrist arthroscopy remains the reference standard for definitive confirmation and staging, while also allowing simultaneous therapeutic intervention. Persistent clinical suspicion despite a negative MRI warrants consideration of diagnostic arthroscopy

To aid clinical interpretation, we added a step-by-step flowchart summarizing the diagnostic pathway for suspected SLIL injury, incorporating the diagnostic performance of standard practice MRI and indicating when arthroscopy should be considered despite a negative MRI result (Fig. 3).

The most important result of the present study is the marked discrepancy between MRI and arthroscopy in the detection of partial lesions. This is clinically relevant because partial SLIL injuries are common and may precede more advanced instability [3]. The scapholunate ligament is anatomically complex, and the thin dorsal, membranous, and palmar components can be difficult to assess on routine static MRI [16]. Arthroscopy remains superior for direct visualization and probing of the ligament, which explains why it continues to serve as the reference standard in most studies and in clinical decision-making [8, 9].

Our findings are consistent with the broader literature showing that MRI performance depends on technical and interpretive factors. Higher field strength and improved sequence optimization can improve depiction of wrist ligaments, and 3-T imaging has been shown to provide better visibility of intercarpal ligaments than 1.5-T imaging [17, 18]. However, our dataset reflects standard practice clinical care in a predominantly rural healthcare setting in Germany, where access to 3-T MRI is still limited and such examinations are rarely performed in standard practice. In parallel, contrast-enhanced techniques may increase diagnostic confidence in selected cases, although clinical MRI still varies widely in quality and protocol design [19]. The present data are especially relevant because they reflect standard practice rather than a narrowly standardized research protocol.

The clinical implication is that a negative MRI should be interpreted cautiously when suspicion for SLIL injury remains high. Persistent pain, mechanical symptoms, or clinical signs of scapholunate instability should prompt further evaluation, and arthroscopy should be considered when definitive structural assessment is needed. MRI remains valuable for identifying alternative causes of wrist pain and for detecting clearly advanced tears, but its limitations must be recognized in partial or dynamic lesions. In that sense, MRI should be regarded as complementary to, rather than a replacement for, arthroscopy [9, 15].

Several limitations should be considered. First, the retrospective design introduces selection bias, because only patients who proceeded to arthroscopy were included. Second, MRI examinations were performed in standard practice with heterogeneous protocols and variable scanner settings, which may have influenced diagnostic performance. However, this approach was chosen deliberately to reflect real-world clinical care. Restricting the analysis to highly standardized MRI examinations from a single radiology centre might have increased internal consistency but would have reduced the generalizability of the findings to everyday practice across different institutions and imaging settings. Third, the interval between MRI and surgery was not uniform, and progression of pathology cannot be excluded in some patients. Fourth, arthroscopic grading remains operator dependent. Previous studies have shown that interobserver agreement is only fair to moderate for several arthroscopic wrist findings, while intraobserver agreement is generally better but still imperfect, indicating that diagnostic variability is also present at the level of the gold standard itself. This limitation should be kept in mind when interpreting the present results, especially because similar variability is well known for wrist MRI as well [20–22]. Finally, missing data in a subset of examinations should be acknowledged, although the cohort size was large enough to support robust conclusions.

## Conclusion

Standard practice wrist MRI shows high specificity but limited sensitivity for scapholunate interosseous ligament lesions when compared with arthroscopy. In this cohort, MRI detected intact ligaments reliably, but a relevant proportion of partial and complete tears remained unrecognized.

These findings support the use of wrist MRI as an adjunctive diagnostic tool; however, its ability to exclude SLIL injury may be limited in non-specialized clinical settings [9, 15]. Persistent clinical suspicion despite negative MRI findings should therefore prompt consideration of arthroscopic evaluation, which remains the reference standard for definitive diagnosis and staging [23].

## Data Availability

No datasets were generated or analysed during the current study.

## Abbreviations

CI: Confidence interval
MRI: Magnetic resonance imaging
NPV: Negative predictive value
PPV: Positive predictive value
SD: Standard deviation
SLAC: Scapholunate advanced collapse
SLIL: Scapholunate interosseous ligament
